# Supplementary material for: Optogenetic patterning generates multi-strain biofilms with spatially distributed antibiotic resistance
Source: Nat Commun. 2024 Nov 1;15:9443. doi: 10.1038/s41467-024-53546-1 (PMC11530673; doi:10.1038/s41467-024-53546-1)
Supplement: Supplementary file 1 — Supplementary Information [file 41467_2024_53546_MOESM1_ESM.pdf]

Supplementary Information

Xiaofan Jin and Ingmar H. Riedel-Kruse

Supplementary Note 1 Widefield fluoresence microscopy analysis of successively patterned biofilms

Widefield microscopy fluoresence confirms that green and red strains are partnered orthogonal, yielding distinct G, R, N, and GR regions.

In addition to shorter-term time-lapse confocal microscopy described in the main text, we also performed long-term endpoint growth experiments where biofilms are left to grow undisturbed for 72h at room temperature, then PBS-rinsed to remove planktonic cells, and finally imaged using a wide-field fluorescent microscope. Quantifying the pre-vs. post-growth levels of fluorescence in the GR overlap region where both red and green strains were patterned, we observed that the RFP signal is significantly increased at the 72h endpoint compared to initial measurement (9.7 ± 0.7AU and 56.7 ± 3.0AU for pre- and post-growth respectively, $p = 1.8 \cdot 10^{-7}$, n=4). GFP fluorescent signal exhibited a similar increase (12.4 ± 1.4AU and 38.8 ± 5.1AU for pre- and post-growth respectively, $p = 1.3 \cdot 10^{-4}$, n=4), further confirming that co-culture biofilms patterned using our tool were actively growing and viable. Comparing fluorescence levels of post-growth IC-RFP and SS-GFP biofilm across different ampicillin concentrations, we found that SS-GFP (i.e., resistant strain) biofilm fluorescence was higher at moderate ampicillin concentrations, presumably due to reduced competition from the IC-RFP (i.e., susceptible strain) biofilm as a result of ampicillin presence.

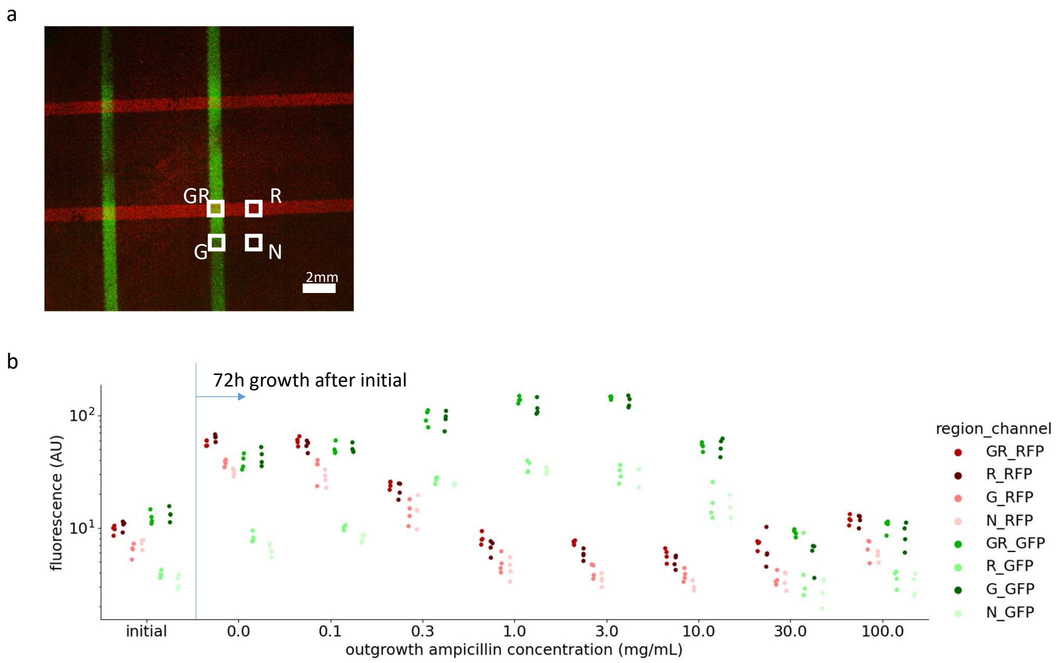

Supplementary Figure 1: **Widefield microscopy analysis confirmed orthogonal patterning. (a)** Image taken using widefield microscopy of multi-patterned biofilm generated using successive patterning with initial IC-RFP biofilm patterned in horizontal stripes, intersected by vertical stripes of the SS-GFP biofilm. Experiment was repeated independently 3 times with similar results. **(b)** Quantification of average fluorescence in patterned regions indicated that GFP signal was high and GR and G regions, and RFP signal was high in GR and R regions, demonstrating orthogonality of patterning between initial IC-RFP biofilm and SS-GFP biofilm. Long-term endpoint measurements indicated that biofilm fluorescent signals for both GFP and RFP strains were increased at 72h compared to 0h, further confirming activity and viability of patterned biofilm communities. Moreover, measurements indicated that signal from amp^R SS-GFP biofilm was higher at moderate ampicillin concentrations (e.g., 0.1-3mg/mL) than with no ampicillin, whereas signal from amp^S IC-RFP biofilm decreased monotonically as ampicillin levels are increased. Patterning was maintained during growth, with GFP signal high in GR and G regions, and RFP signal high in GR and R regions. n=4 IC-RFP + SS-GFP co-cultured biofilms. Source data are provided as a source data file.

Supplementary Note 2 Quantifying biofilm interdigitation between from confocal biofilm images

To estimate a characteristic length scale of the interdigitation between strains, we computed the normalized spatial X-Y cross-correlation function between the RFP and GFP signal in the GR region where both IC-RFP and SS-GFP biofilm are patterned (using the Z-slice mean position between the slices highest overall RFP and GFP signal). We found that this cross-correlation signal has a negative minimum at the origin characteristic of neighbour exclusion. Comparing magnitude of this anticorrelation across growth time and antibiotic concentration, we found that this observed strain-strain exclusion decreased as growth progressed which is indicative of increased intermixing / reduced exclusion, and that this effect depends on the presence of antibiotic for both kanamycin and ampicillin. This is in line with earlier work showing that biofilm community intermixing increases with antibiotic stress [1].

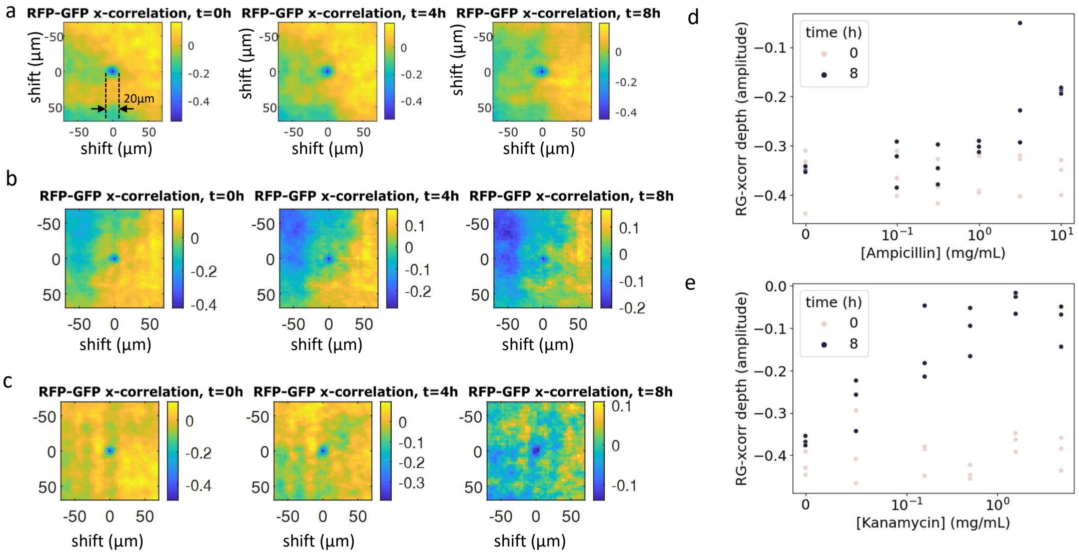

Supplementary Figure 2: **Spatial X-Y cross-correlation between RFP and GFP signal.** **(a)** Cross-correlation between RFP$^+$ and GFP$^+$ signal in GR region at 0, 4 and 8h, no antibiotics; 20µm cross-exclusion zone highlighted. **(b)** Cross-correlation between RFP$^+$ and GFP$^+$ signal in GR region at 0, 4 and 8h, 1mg/mL ampicillin. **(c)** Cross-correlation between RFP$^+$ and GFP$^+$ signal in GR region as at 0, 4 and 8h, 0.5mg/mL kanamycin. **(d)** Depth of minima at origin of cross-correlation between RFP$^+$ and GFP$^+$ signal in GR region as a function of increasing ampicillin concentration. n=3 IC-RFP + SS-GFP co-cultured biofilms. Source data are provided as a source data file. **(e)** Depth of minima at origin of cross-correlation between RFP$^+$ and GFP$^+$ signal in GR region as a function of increasing kanamycin concentration. n=3 IC-RFP + SS-GFP co-cultured biofilms. Source data are provided as a source data file.

Supplementary Note 3 Combined differentiation and successive patterning

We generated biofilms with three distinct phenotypes using a combined approach involving patterning and differentiation with DS-mRuby2, followed by a successive patterning step with SS-GFP. Resulting biofilm demonstrated orthogonal patterning of undifferentiated DS-mRuby2 strain (low mRuby2 expression), differentiated DS-mRuby2 strain (high mRuby2 expression), and SS-GFP strain (GFP expression).

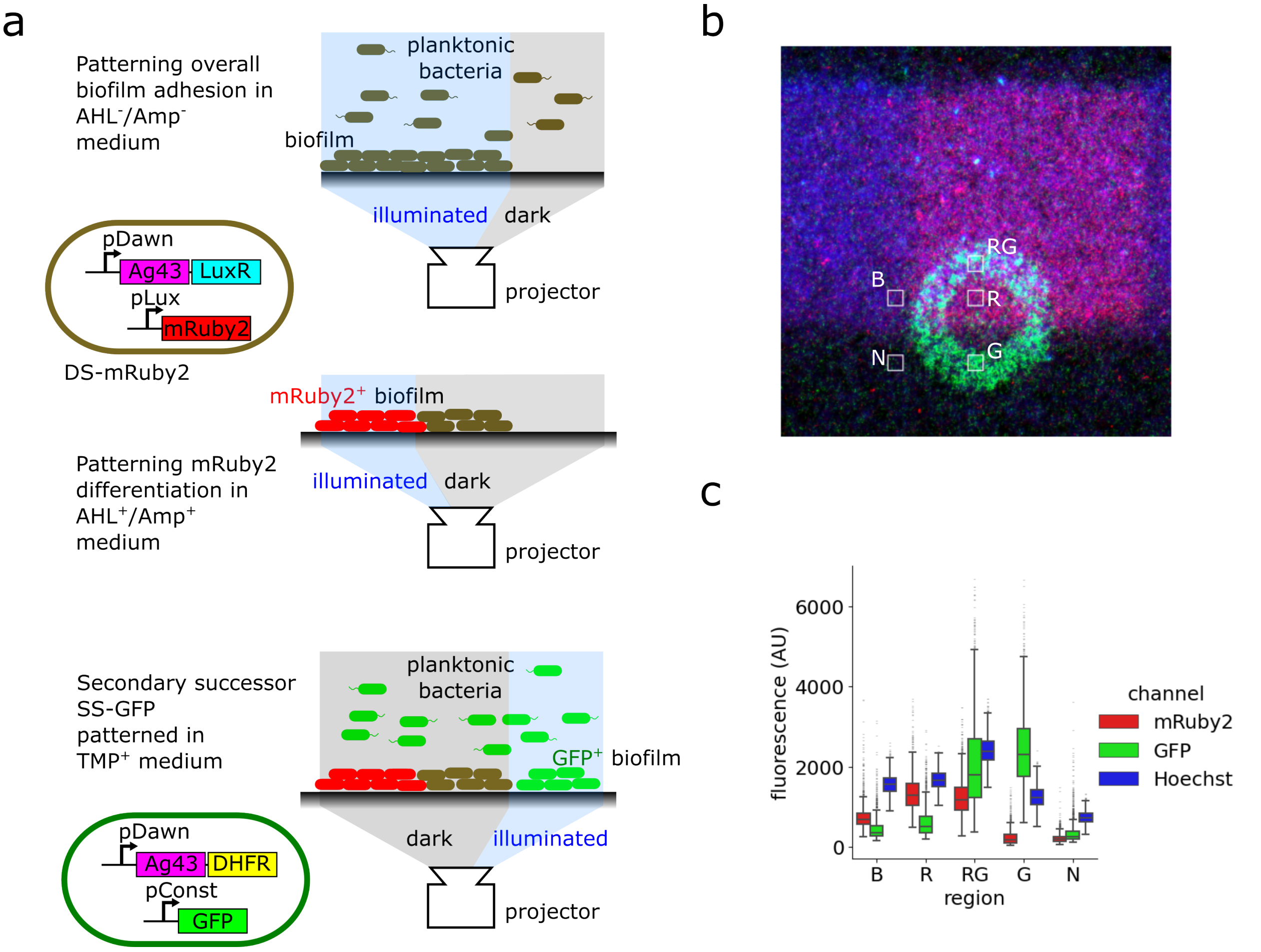

Supplementary Figure 3: **Description and quantification of biofilms patterned using differentiation followed by successive adhesion (a)** Schematic of differentation plus succession protocol: Biofilm of DS-mRuby2 is patterned and differentiated as before. After wash step, SS-GFP strain is introduced and patterned as previously described. **(b)** Annotation of specific regions (40 x 40 pixels each) in image of final biofilm used for quantification of mRuby2, GFP, and Hoescht channels. **(c)** Quantification of mRuby2, GFP, and Hoescht channels in each region marked in (b), confirming that as expected mRuby2 channel is highest in R and RG regions where DS-mRuby2 was differentiated to express mRuby2, GFP was highest in RG and G regions where SS-GFP was patterned, and Hoechst was high in all regions except N where no biofilm was patterned. n=1600 pixels, data are presented as mean +/- SD across all pixels in each region. Source data are provided as a source data file.

Supplementary Note 4 Quantification and statistical comparison of IC-RFP and SS-GFP biofilm expansion

We tracked the extent to which the biofilm boundary has expanded after up to 10h of culture using confocal microscopy.

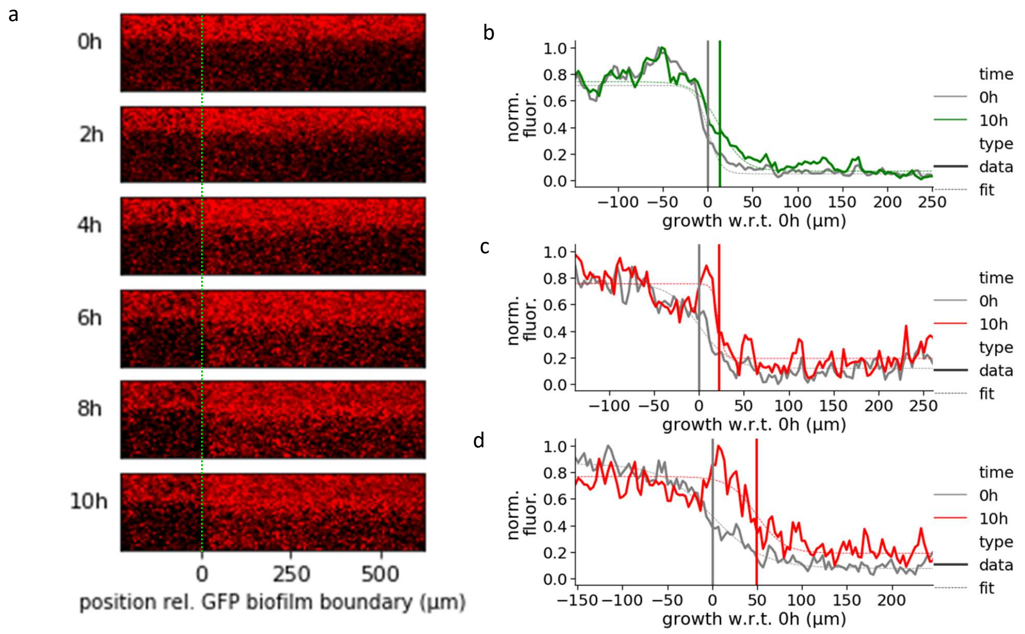

Supplementary Figure 4: **Outward expansion of SS-GFP and IC-RFP biofilm over time. (a)** Representative example of outward expansion of IC-RFP biofilm at the growth front without ampicillin (analogous to Fig. 3c in main text but for IC-RFP instead of SS-GFP biofilm). Dotted green line marks initial boundary location of SS-GFP biofilm. Experiment was repeated independently 3 times with similar results. **(b)** Representative curve-fitting of outward expansion of SS-GFP biofilm at the growth front at position -150µm without ampicillin (analogous to Fig. 3d in main text but for -150µm instead of 400µm position) Grey and green vertical bars indicate extent of the expanding biofilm front inferred from fits at 0h and 10h respectively. **(c)** Representative curve-fitting of outward expansion of IC-RFP biofilm at the growth front at position -150µm without ampicillin. Grey and red vertical bars indicate extent of the expanding biofilm front inferred from fits at 0h and 10h respectively. **(d)** Representative curve-fitting of outward expansion of IC-RFP biofilm at the growth front at position 400µm without ampicillin. Grey and red vertical bars indicate extent of the expanding biofilm front inferred from fits at 0h and 10h respectively.

In the absence of ampicillin, both IC-RFP and SS-GFP biofilms exhibited clear outward expansion over the 10h interval, with greater expansion for biofilms patterned away from their counterpart strain. We found during this 10h interval, IC-RFP and SS-GFP biofilms patterned 400µm from the boundary of their counterpart strain exhibit growth rates of $4.3 \pm 0.4 \mu m/h$ and $4.5 \pm 0.3 \mu m/h$ (ordinary least squares p =

$5.3 \cdot 10^{-12}$, n=3) respectively. Both IC-RFP and SS-GFP growth rates were significantly greater than zero (ordinary least squares p = $2.1 \cdot 10^{-9}$ and p = $5.3 \cdot 10^{-12}$ respectively, n=3)

Comparing different spatial positions relative to the red-green overlap region (GR), we discovered that expansion of biofilm was more limited in overlap regions at position -150μm relative to the boundary of the counterpart strain. At this position, where biofilm was expanding into space already colonized by the counterpart strain, IC-RFP and SS-GFP biofilms exhibited growth rates of $2.2 \pm 0.4 \mu m/h$ and $1.9 \pm 0.2 \mu m/h$ respectively. While these -150μm position growth rates were still significantly greater than zero (ordinary least squares p = $2.5 \cdot 10^{-5}$ and $2.0 \cdot 10^{-7}$ for IC-RFP and SS-GFP strains respectively, n=3), growth was significantly less than that measured at the 400μm position, which we confirmed using a linear mixed model with position and time treated as fixed and random effects respectively (p = $8.1 \cdot 10^{-5}$ and $1.5 \cdot 10^{-13}$ for IC-RFP and SS-GFP strains respectively, n=3 replicates, 5 time-groups of 2, 4, 6, 8, 10h).

When ampicillin was introduced, we observed decreased growth of susceptible IC-RFP strain. For instance at 1mg/mL ampicillin, in the +400μm position from boundary, the IC-RFP biofilm growth rate was no longer significantly greater than zero ($0.4 \pm 0.4 \mu m/h$ ordinary least squares p = 0.32) while the SS-GFP biofilm growth rate remained significantly greater than zero ($0.4 \pm 0.4 \mu m/h$ ordinary least squares p = 0.32). At 10mg/mL ampicillin, significant growth was not observed in the +400μm position from boundary for either IC-RFP ($0.2 \pm 0.2 \mu m/h$ ordinary least squares p = 0.29) or SS-GFP ($0.2 \pm 0.1 \mu m/h$ ordinary least squares p = 0.14).

To more closely investigate this effect, we focused on growth of co-culture biofilms at different spatial positions besides the +400μm position. We observed that ampicillin-based growth inhibition of susceptible IC-RFP biofilm – while still present – was noticeably damped at the position = 0μm (i.e., on boundary of SS-GFP resistant biofilm). Growth extent of IC-RFP susceptible biofilm at position = 0μm remained significantly greater than zero ($2.3 \pm 0.2 \mu m/h$ ordinary least squares p = $9.8 \cdot 10^{-10}$) at 1mg/mL ampicillin. At this concentration, growth of susceptible IC-RFP biofilms at position = 0μm was greater than that observed at either position = +400μm ($0.4 \pm 0.4 \mu m/h$) or position = -150μm (0.6 ± 0.2μm), which we confirmed using a linear mixed model with position and time treated as fixed and random effects respectively (p = $6.1 \cdot 10^{-9}$ relative to position = +400$*upmu*m and p = $8.1 \cdot 10^{-6}$ relative to position = -150μm, n=3 replicates, 5 time-groups of 2, 4, 6, 8, 10h).

This was in contrast with the no-ampicillin case where IC-RFP biofilm growth rate at the boundary position 0μm ($4.4 \pm 0.3 \mu m/h$) was significantly greater than that at the -150μm position ($2.2 \pm 0.4 \mu m/h$) but not significantly greater than that at the +400μm position ($4.3 \pm 0.4 \mu m/h$), which we confirmed using a linear mixed model with position and time treated as fixed and random effects respectively (p = $1.6 \cdot 10^{-6}$ relative to position = -150$*upmu*m and p = 0.68 relative to position = +400μm, n=3 replicates, 5 time-groups of 2, 4, 6, 8, 10h). At 10mg/mL ampicillin, significant zon-zero growth was not observed for the IC-RFP biofilm at any position ($0.1 \pm 0.4 \mu m/h$ ordinary least squares p = 0.78 for position -150μm, $0.1 \pm 0.2 \mu m/h$ ordinary least squares p = 0.75 for position 0μm, $0.2 \pm 0.2 \mu m/h$ ordinary least squares p = 0.29 for position +400μm). Collectively, this demonstrated that this apparent protective effect – where nearby SS-GFP biofilms could support growth in otherwise susceptible IC-RFP biofilms – was most pronounced at moderate ampicillin concentrations around 1mg/mL ampicillin. In other words, our results indicate that at moderate ampicillin concentrations around 1mg/mL, growth of susceptible biofilm is severely inhibited unless they are co-cultured with resistant biofilms, and that this shared protective effect is most pronounced when susceptible cells are in close proximity to resistant biofilm.

Analyzing these results from the point of view of SS-GFP resistant biofilm, we observed that these cells were capable of growing at ampicillin concentration up to 3mg/mL with growth rate for the +400μm position measured at $4.7 \pm 0.6 \mu m/h$, compared with $4.5 \pm 0.3 \mu m/h$ without ampicillin. Eventually, clear inhibitive effects were observed at 10mg/mL, where growth rate is reduced to $0.2 \pm 0.1 \mu m/h$. Using a linear mixed model with antibiotic level and time treated as fixed and random effects respectively, we confirmed that increasing from 0mg/mL to 3mg/mL ampicillin had only a weakly significant effect on growth (p = 0.034, n=3 replicates, 5 time-groups of 2, 4, 6, 8, 10h), while increasing from 0mg/mL to 10mg/mL ampicillin had a strongly significant effect (p = $1.7 \cdot 10^{-16}$, n=3 replicates, 5 time-groups of 2, 4, 6, 8, 10h) Notably, for the SS-GFP biofilm adjacent to IC-RFP biofilms (position = 0μm, i.e., on boundary), the addition of a low 0.1mg/mL concentration of ampicillin significantly improved growth rate relative to no ampicillin condition ($3.4 \pm 0.2 \mu m/h$ vs. $2.3 \pm 0.2 \mu m/h$ respectively), which we confirmed using a linear mixed model with antibiotic level and time treated as fixed and random effects respectively (p = $1.0 \cdot 10^{-8}$). Consistent with this

confocal time-lapse finding, endpoint measurements of red and green fluorescence in the GR biofilm region (both strains present) indicated that signal from resistant SS-GFP biofilm was higher at low-to-moderate ampicillin concentrations (e.g., 0.1-3mg/mL) than with no ampicillin, whereas signal of susceptible IC-RFP biofilm decreased monotonically as ampicillin levels had increased (Supplemental Section S1). These results indicate that addition of increasing ampicillin not only inhibits growth of susceptible IC-RFP cells, but also improves growth of resistant SS-GFP biofilm, consistent with an underlying competition for shared nutrients.

Supplementary Note 5 PBS controls of IC-RFP and SS-GFP biofilm growth

To determine that outward expansion of biofilm over time is not due simply to biomass outward diffusion, we tracked biofilm expansion in cultures with PBS instead of culture medium. These PBS controls did not exhibit any outward expansion.

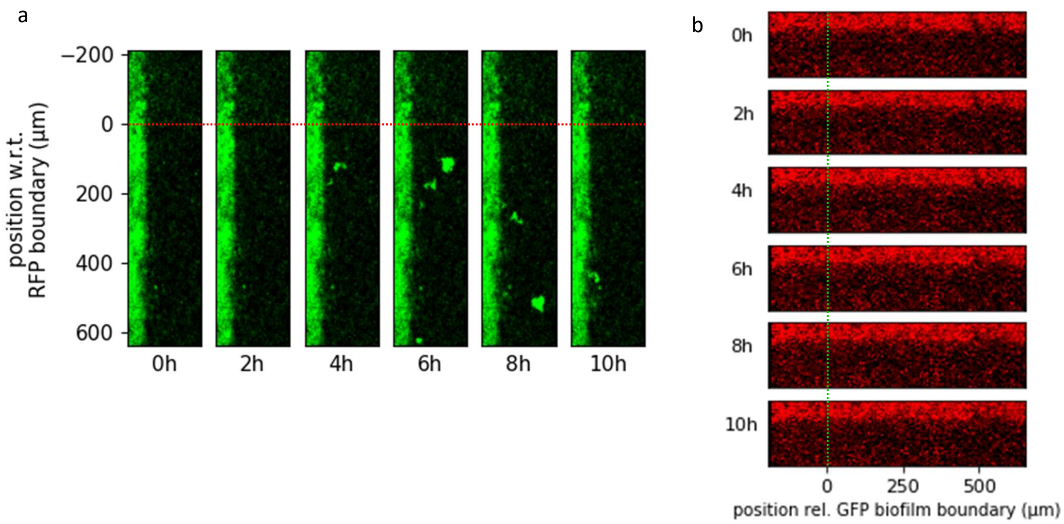

Supplementary Figure 5: **Patterned biofilms left in PBS instead of culture medium do not exhibit outward growth.** **(a)** Representative example of lack of outward expansion of SS-GFP biofilm for PBS control. Dotted red line marks initial boundary location of IC-RFP biofilm. Experiment was repeated independently 3 times with similar results **(b)** Representative example of lack of outward expansion of IC-RFP biofilm for PBS control. Dotted green line marks initial boundary location of SS-GFP biofilm. Experiment was repeated independently 3 times with similar results

Supplementary Note 6 Single strain controls of IC-RFP and SS-GFP biofilm growth

To assess the effects of co-culture context on biofilm growth, we ran a control where the two strains used for successive patterning were individually patterned alone in a single-strain context. We found that in the absence of ampicillin, the growth extent of strains was improved when grown as single-strain compared to co-culture, indicative of competitive effects in co-culture. For instance, in cultures without any ampicillin present, IC-RFP and SS-GFP monoculture biofilms exhibited growth rates of 8.4 ± 0.6µm/h and 33.1 ± 1.4µm/h respectively up to the 8h timepoint. These growth rates were significantly higher than those observed in co-culture biofilms (4.2 ± 0.5µm/h and 5.0 ± 0.3µm/h respectively for IC-RFP and SS-GFP), which we confirmed using a linear mixed model with culture condition (mono- vs. co-culture) and time treated as fixed and random effects respectively ($p = 6.1 \cdot 10^{-4}$ and $9.7 \cdot 10^{-10}$ for IC-RFP and SS-GFP strains respectively, n=3 replicates, 4 time-groups of 2, 4, 6, 8h). Note that due to overgrowth of planktonic cells in the single strain monoculture biofilms, analysis was limited up to the 8h timepoint for single strain cultures.

Inhibition of IC-RFP strain growth at low to moderate levels of ampicillin was noticeably more pronounced in the single strain context compared to co-culture. In single strain susceptible IC-RFP biofilm where resistant cells were not present, growth rate decreased from 8.4 ± 0.6µm/h with no ampicillin, to -0.6 ± 0.2µm/h at 0.1mg/mL ampicillin, effectively meaning that the lowest concentration of ampicillin we tested fully abrogated growth of single strain susceptible IC-RFP biofilm. By contrast, this decrease was less dramatic for IC-RFP in co-culture biofilms even at the +400µm position (away from the SS-GFP resistant biofilm) which went from 4.2 ± 0.5µm/h with no ampicillin, to 2.3 ± 0.4µm at 0.1mg/mL ampicillin. Using a linear mixed model with culture condition (mono- vs. co-culture) and time treated as fixed and random effects respectively, we confirmed that at 0.1mg/mL ampicillin, growth of IC-RFP biofilm is significantly faster in co-culture than monoculture ($p = 2.8 \cdot 10^{-7}$ n=3 replicates, 4 time-groups of 2, 4, 6, 8h). These single IC-RFP susceptible biofilm cultures further confirm that the ability of RFP biofilm to grow / expand at low-moderate ampicillin concentrations (i.e.,0.1-1mg/mL) does in fact depend on presence of resistant SS-GFP strain.

These comparisons with single-strain biofilms should be interpreted cautiously due to subtle differences in the patterning protocol, as single-strain IC-RFP is not exposed to secondary effects of SS-GFP patterning step, and single-strain SS-GFP biofilm patterning occurs on a completely untouched plastic surface, compared with plastic that had been pre-exposed to IC-RFP cells. For instance, treatment with TMP during dual-strain biofilm formation might improve phenotypic resistance to Amp in a growth rate-dependent manner, since different populations have been growing / non-growing for different amounts of time. These factors likely have some effect on the adhesion efficiency of the initially patterned biofilms.

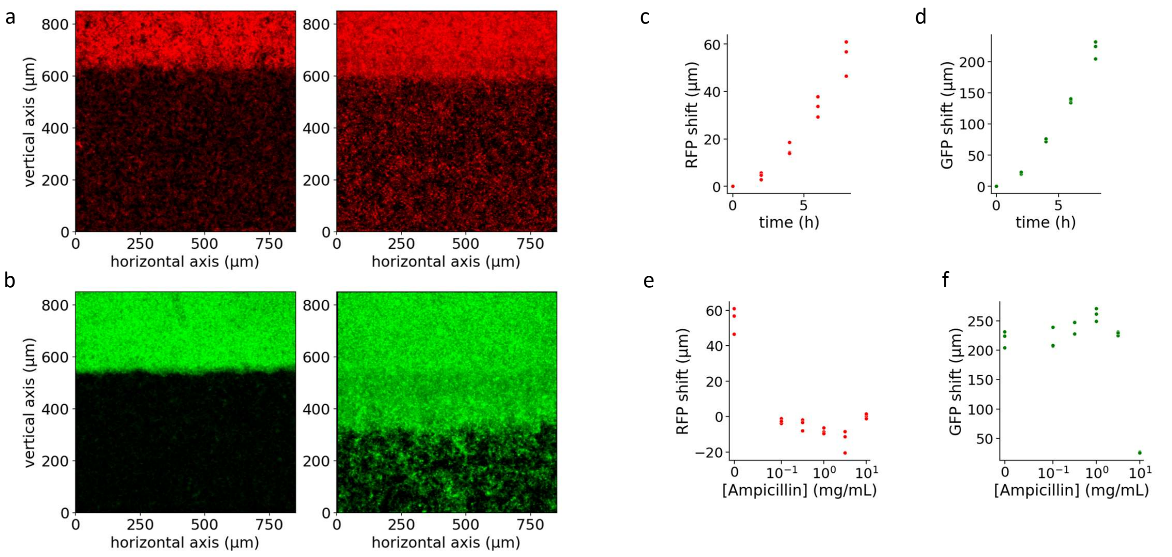

Supplementary Figure 6: **Monoculture controls of SS-GFP and IC-RFP biofilms.** **(a)** Representative example of outward expansion of monoculture IC-RFP biofilm (0h on the left, 8h on the right). Experiment was repeated independently 3 times with similar results. **(b)** Representative example of outward expansion of monoculture SS-GFP biofilm (0h on the left, 8h on the right). Experiment was repeated independently 3 times with similar results. **(c)** Outward expansion of monoculture IC-RFP biofilm over time without ampicillin. n=3 monoculture IC-RFP biofilms. Source data are provided as a source data file. **(d)** Outward expansion of monoculture SS-GFP biofilm over time without ampicillin. n=3 monoculture SS-GFP biofilms. Source data are provided as a source data file. **(e)** Growth extent of IC-RFP biofilm at 8h as a function of ampicillin concentration. Note immediate drop-off indicating lack of growth even at lowest ampicillin concentration of 0.1mg/mL. n=3 monoculture IC-RFP biofilms. Source data are provided as a source data file. **(f)** Growth extent of SS-GFP biofilm at 8h as a function of ampicillin concentration. SS-GFP biofilm remains resistant up until $10mg/mL$. n=3 monoculture SS-GFP biofilms. Source data are provided as a source data file.

Supplementary Note 7 Parameters for computational model of biofilm growth

We used the following initial conditions (Supplementary Table 1) and parameters for the model (Supplementary Table 2). Simulations were run on a 64 by 64 pixel grid to simulate the 850μm by 850μm growth area in the real-world confocal imaging data. The model was run using time steps of 6s for 6000 steps effectively representing 10h of growth.

Init. cond.	Dim.	Description	Value(s)	Notes
ρ_{nut0}	M/L^3	starting nutrient concentration	2mg/mL	Based on glucose concentration in M63 culture medium
c_{amp0}	N/L^3	starting ampicillin concentration	0, 0.29, 0.9, 2.9, 9, and 28.6mM	Based on tested ampicillin concentrations in M63 culture medium of 0, 0.1, 0.32, 1, 3.16, and 10mg/mL and ampicillin MW of 350g/mol
$c_{blaMedia0}$, $c_{blaBiofilm0}$	N/L^3	starting beta-lactamase concentrations	0	Assuming no initial beta-lactamase
b_{R0},b_{G0}	M/L^2	Average starting 2D cellular biomass density (dry mass)	3pg/μm^2 in illuminated regions (top quarter of simulation grid for red cells, left quarter of simulation grid for green cells), 0.03pg/μm^2 in un-illuminated regions	Based on maximum biofilm thickness estimate of 200 - 250μm, assuming 130.4 kg/m^3 biofilm dry mass density[2], and 10% cellular biomass proportion[3]; Assumes 1% leakiness in un-illuminated regions.

Supplementary Table 1: **Initial conditions used for modeling as determined from experimental setup.**

Param.	Dim.	Description	Value(s)	Notes
K_{nut}	M/L^3	Monod growth affinity constant	0.004mg/mL	Experimentally estimated for E. coli growing on glucose [4, 5]
μ_{max}	1/T	Maximum growth rate	$4.8 \cdot 10^{-5}s^{-1}$	Estimated doubling time for E. coli as biofilm based on crystal violet growth curves of approximately 4h [6]
b_{max}	M/L^2	Maximum (i.e. carrying capacity) 2D biomass density, dry mass	6pg/μm^2	Rough estimate based on b_{R0}, b_{G0} of patterned biofilm of 3pg/μm^2, and approximate observed 2x fluorescence intensity increase between initially patterned biofilms and final biofilms after growth
γ_{bm}	1/T	First order biomass self degradation constant	$5.6 \cdot 10^{-6}s^{-1}$	Using measured K-12 death rate on glucose of 0.48day^{-1} [7]

D_{bm}	L^2/T	Diffusivity of biomass	$0.149 \mu m^2/s$	Estimated using parameter search, fitting to experimental data (see Supplemental Section Supplementary Note 8)
n	1	Hill coefficient for ampicillin inhibition	0.75	Using experimentally measured value [8]
K_{ampR}	N/L^3	Half-max hill constant for ampicillin inhibition of susceptible red cells	$2.7 \mu M$	Using observed MIC for $ampC^-$ DH5alpha E. coli[9]
K_{ampG}	N/L^3	Half-max hill constant for ampicillin inhibition of resistant green cells	$1379 \mu M$	Using observed MIC for $ampC^+$ DH5alpha E. coli[9]
k_{catBla}	$1/T$	Catalysis coefficient for beta lactamase degradation of ampicillin	$4.2 s^{-1}$	Using measured parameters for E. coli K-12 [10]
K_{dBla}	N/L^3	Hill coefficient for beta lactamase degradation of ampicillin	$3.5 \mu M$	Using measured parameters for E. coli K-12 [10]
γ_{amp}	$1/T$	First order ampicillin self degradation constant	$7.4 \cdot 10^{-6} s^{-1}$	Using pH 7.0 measured value of $0.02681 h^{-1}$ [11]
p_{bla}	$\frac{\text{molecule}}{\text{cell} \cdot T}$	Molecular beta lactamase production rate per cell	$0.013 \frac{\text{molecules}}{\text{cell} \cdot s}$	Estimated using parameter search, fitting to experimental data (see Supplemental Section Supplementary Note 8)
m_{cell}	M/cell	dry cellular biomass per bacterial cell	0.3pg/cell	Assuming wet biomass of 1pg/cell, with 30% dry fraction [12]
N_A	molecule/N	Avogadro's constant	$6.02 \cdot 10^{23}$ molecules/mole	used to convert beta lactamase production rate from molecular to molar basis
γ_{bla}	$1/T$	First order beta lactamase self degradation constant	$9.6 \cdot 10^{-6} s^{-1}$	Assuming typical approximate protein half life of 20h [13]
Y	1	Nutrient to dry biomass yield ratio	0.316	Estimated for E. coli growing in M63 culture medium[14]
h	L	Height of culture media	2mm	Calculated based on 2mL culture volume in 6-well plate surface area of $10 cm^2$
D_{amp}	L^2/T	Diffusivity of ampicillin in culture medium	$400 \mu m^2/s$	Experimental value [15]. As this greatly exceeds diffusivity of biomass, numerical model implementation applies separation of timescales and assumes this is instantaneously averaged across simulation grid in each timestep

k_{eq}	1/T	Rate-constant of equilibration of ampicillin between biofilm and culture medium	$2.86 \cdot 10^{-5}$/s	Estimated using parameter search, fitting to experimental data (see Supplemental Section Supplementary Note 8)
D_{nut}	L^2/T	Diffusivity of nutrients	600μm^2/s	Using measured value of glucose diffusivity in water [16]. As this greatly exceeds diffusivity of biomass, numerical model implementation applies separation of timescales and assumes this is instantaneously averaged across simulation grid in each time-step

Supplementary Table 2: **Parameters used for modeling as determined from experimental setup, literature, or parameter search fitting to experimental data.**

Supplementary Note 8 Parameter search for biomass diffusivity, beta-lactamase production rate, and culture medium-to-biofilm ampicillin equilibration rate

Parameter search for biomass diffusivity D_bm was conducted by numerically simulating 10h of growth and tracking simulated biofilm growth, using no ampicillin concentration, testing 201 evenly log-spaced D_bm values between 10^{-2} to 10^{0}µm^2/s. For each value of D_bm, the extent of simulated biofilm growth between positions boundary-150µm and boundary +400µm were calculated at 50µm intervals in space and 2h intervals in time between t=0h to t=10h, for both red and green biomass (representing experimental IC-RFP and SS-GFP biofilm respectively) Mean discrepancy between experimental and simulated growth extents at these times and positions was compared across tested D_bm values. By fitting a cubic radial basis function of discrepancies for these 201 tested values (implemented using scipy.interpolate.RBFInterpolator) to describe mean discrepancy as a function of D_bm, we found the optimal value of D_bm that minimized discrepancy with experimental findings to be 0.149µm^2/s. To generate an estimated range for the fitted parameter value of D_{bm}, we applied a bootstrap approach where we generated 1000 sets 'simulated' experimental observations by drawing growth extents from a random normal distribution parameterized by the observed mean and standard deviation at each timepoint and position. We then took the middle 95% of the distribution of D_{bm} values that minimized these 1000 simulated sets as the estimated range, yielding a 95% CI range of 0.132µm^2/s - 0.164µm^2/s.

After determining the value of D_{bm}, a similar approach was used to determine the optimal values of beta-lactamase production rate (p_{bla}), and culture medium-to-biofilm ampicillin equilibration rate (k_{eq}), in this case testing 301 evenly log-spaced p_{bla} values between $8.7 \cdot 10^{-5}$molecules/cell/s to $8.7 \cdot 10^{-2}$molecules/cell/s, alongside 301 evenly log-spaced k_{eq} values between $1 \cdot 10^{-3}$/s to $1 \cdot 10^{-6}$/s for a total of 90601 value pairs. For each value-pair of p_{bla}, k_{eq}, the extent of simulated biofilm growth between positions boundary-150µm and boundary+400µm were calculated at 50µm intervals in space and 2h intervals in time between t=0h to t=10h, with starting ampicillin concentrations c_{amp0} of 0, 0.29, 0.9, 2.9, 9, and 28.6mM (i.e. 0, 0.1, 0.32, 1, 3.16, and 10mg/mL) for both red and green biomass (representing IC-RFP and SS-GFP respectively). Mean discrepancy between experimental and simulated growth extents at these times, positions, and ampicillin concentrations were compared across tested p_{bla}, k_{eq} value pairs. By 2-D cubic spline interpolation (implemented using scipy.interpolate.RegularGridInterpolator) of discrepancies over tested $log(p_{bla})$ and $log(k_{eq})$ values, we found the optimal value of p_{bla} to be 0.013molecules/cell/s (0.95% CI range 0.008 - 0.014molecules/cell/s) and k_{eq} to be 2.86 $\cdot 10^{-5}$s^{-1} (0.95% CI range 1.72 - 3.12 $\cdot 10^{-5}$s^{-1}), to minimize discrepancy with experimental findings.

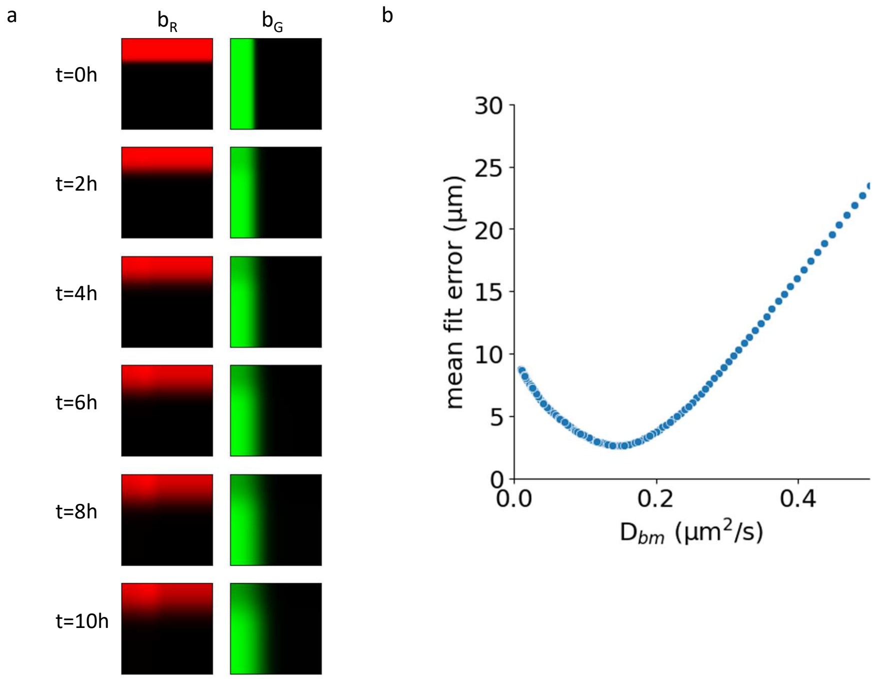

Supplementary Figure 7: **Details on numerical model implementation and parameter search. (a)** Representative results from numerical model simulation over 10h with 1mg/mL ampicillin as starting condition. **(b)** Results of parameter search for optimal biomass diffusivity (D_{bm}), seeking value that minimizes discrepancy of biofilm growth extents relative to experimental results in cultures without ampicillin present.

Supplementary Note 9 Comparison with kanamycin in place of ampicillin antibiotic

To compare antibiotic sharing effects of ampicillin with a non-shared protection counterpart, we performed a parallel series of experiments with kanamycin. Whereas the mechanism of action for beta-lactam antibiotics such as ampicillin is inhibition of cell wall peptidoglycan biosynthesis, kanamycin is an aminoglycoside antibiotic, which works by disrupting intracellular protein synthesis, via ribosomal binding [17]. The resistance gene in kanR strains (BioBrick BBa_P1003) encodes for aminoglycoside kinase [18], which inactivates intracellular kanamycin via phosphorylatation [19]. Given the intracellular nature of kanamycin attack and resistance, there is no expectation for kanaycin resistance effects to be shared between cells, unlike beta lactam resistance which occurs in an extracellular context and whose shared effects have been reported multiple times [20, 1, 21].

Using the same initial patterning as with the ampicillin culture experiments (Fig. 4), we repeated the same series of post-patterning cultures across a wide range of kanamycin concentrations starting from the typical inhibitory concentration used for planktonic cell culture (0.05mg/mL) [18] to 5mg/mL. In these experiments, the same successive adhesion strains were used as before, but now the IC-RFP strain was the resistant strain, as it carried the aminoglycoside kinase gene, and the SS-GFP strain was the susceptible one. We observed many similarities with the ampicillin experiments, including outward growth over time of the biofilms, which was fastest in the outer boundary+400μm position when no kanamycin is present. However, unlike the ampicillin experiments, we did not find evidence of spatially mediated shared antibiotic protection, i.e., failing to observe a zone of protection at any concentration of kanamycin wherein the susceptible SS-GFP cells near the resistant IC-RFP cells grow better than those farther away from resistant IC-RFP cells. This aligns with expectations due to intracellular nature of resistance mechanism for kanamycin resistance[19].

Using our diffusive growth modelling framework, we simulated growth of co-culture biofilm where red biomass was resistant to kanamycin (kan^R) and green biomass was susceptible (kan^S). Unlike the ampicillin model, resistant biomass in this updated model do not produce enzyme to degrade kanamycin, thus they do not confer any benefit to susceptible biomass. These simulations use biophysically realistic parameters, specifically changing K_{ampR} and K_{ampG} from the ampicillin model to the analogous K_{kanR} and K_{kanG} describing Half-max hill constant for kanamycin inhibition of experimental IC-RFP (kan^R) and SS-GFP (kan^S) biofilm respectively. We used K_{kanR}=10.3mM and K_{kanG}=0.033mM based on literature values of minimum inhibitory concentration (MIC) in K-12 E. coli with aminoglycoside-kinase of 5mg/mL (using the MIC for related amikacin antibiotic [22]) and MG1655 E. coli without aminoglycoside-kinase of 0.016mg/mL [23], with kanamycin molecular weight of 484.5 g/mol.

Simulation results align with experimental findings, and we did not observe a zone of protection at any concentration of kanamycin wherein the susceptible SS-GFP cells near the resistant IC-RFP cells grow better than those farther away from resistant IC-RFP cells. This lack of spatially mediated shared kanamycin protection aligns with expectations due to the intracellular nature of the resistance mechanism for kanamycin resistance[19].

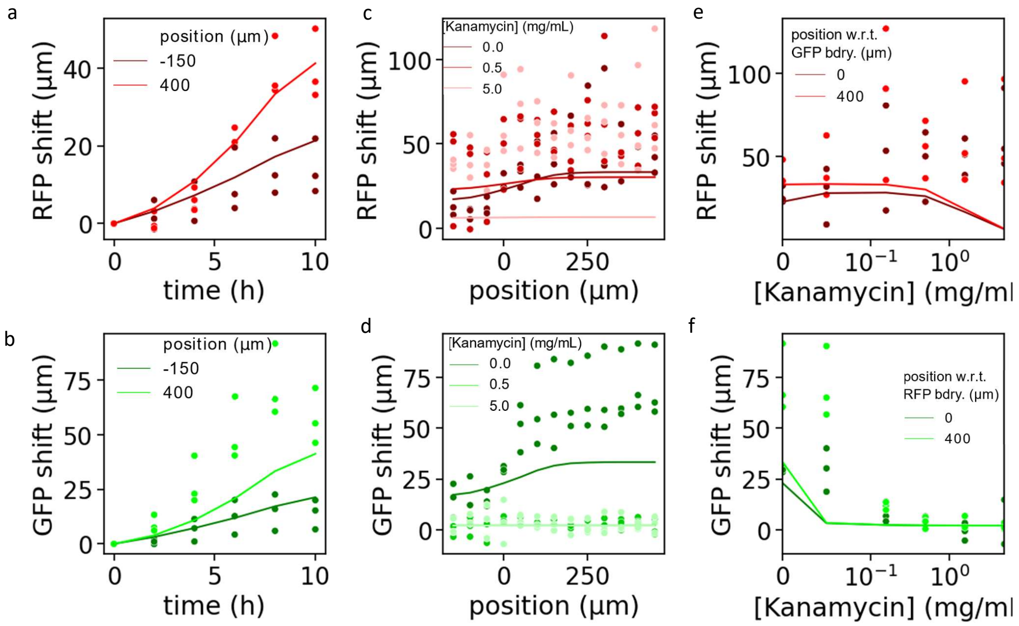

Supplementary Figure 8: **No antibiotic-protection sharing observed with kanamycin experiments. Source data are provided as a source data file.** **(a)** Tracking growth of IC-RFP biofilm over time in no-kanamycin culture. Experimental results (n=3 IC-RFP + SS-GFP co-cultured biofilms) were corroborated by numerical predictions of biophysical model (solid lines). (Analogous to Fig. 3e in main text for ampicillin experiments.) **(b)** Tracking growth of SS-GFP biofilm over time in no-kanamycin culture. Experimental results (n=3 IC-RFP + SS-GFP co-cultured biofilms) were corroborated by numerical predictions of biophysical model (solid lines). (Analogous to Fig. 3f in main text for ampicillin experiments.) **(c)** Growth extent at 8h of kan-resistant IC-RFP biofilm as a function of position relative to boundary of SS-GFP biofilm. Experimental results (n=3 IC-RFP + SS-GFP co-cultured biofilms) were corroborated by numerical predictions of biophysical model (solid lines). (Analogous to Fig. 4c in main text for ampicillin experiments.) **(d)** Growth extent at 8h of kan-susceptible SS-GFP biofilm as a function of position relative to boundary of IC-RFP biofilm. Experimental results (n=3 IC-RFP + SS-GFP co-cultured biofilms) were corroborated by numerical predictions of biophysical model (solid lines). (Analogous to Fig. 4e in main text for ampicillin experiments. Note the lack of a zone of protection as seen in Fig. 4c for amp-susceptible IC-RFP biofilm growth.) **(e)** Growth extent at 8h of kan-resistant IC-RFP biofilm as a function of kanamycin concentration. Experimental results (n=3 IC-RFP + SS-GFP co-cultured biofilms) were corroborated by numerical predictions of biophysical model (solid lines). (Analogous to Fig. 4b in main text for ampicillin experiments.) **(f)** Growth extent at 8h of kan-susceptible SS-GFP biofilm as a function of kanamycin concentration. Experimental results (n=3 IC-RFP + SS-GFP co-cultured biofilms) were corroborated by numerical predictions of biophysical model (solid lines). (Analogous to Fig. 4d in main text for ampicillin experiments.)

Supplementary References

[1] Anupama Sharma and Kevin B. Wood. Spatial segregation and cooperation in radially expanding microbial colonies under antibiotic stress. *The ISME Journal*, 15(10):3019–3033, 2021.

[2] S Sabarunisha Begum and K V Radha. Comparative Kinetic Studies and Performance Evaluation of Biofilm and Biomass Characteristics of Pseudomonas fluorescens in Degrading Synthetic Phenolic Effluent in Inverse Fluidized Bed Biofilm Reactor. *Water Environment Research*, 88(5):415–424, dec 2016.

[3] Hans-Curt Flemming and Jost Wingender. The biofilm matrix. *Nature Reviews Microbiology*, 8(9):623–33, aug 2010.

[4] Jacques Monod. *Recherches sur la croissance des cultures bacteriennes.* Hermann and Cie Paris, Paris SE - 1 preliminary leaf, 210 pages 1 leaf including tables, diagrams 25 cm, 1942.

[5] Heinrich Senn, Urs Lendenmann, Mario Snozzi, Geoffrey Hamer, and Thomas Egli. The growth of Escherichia coli in glucose-limited chemostat cultures: a re-examination of the kinetics. *Biochimica et Biophysica Acta (BBA) - General Subjects*, 1201(3):424–436, 1994.

[6] Maritrini Colón-González, M.Marcela Méndez-Ortiz, and Jorge Membrillo-Hernández. Anaerobic growth does not support biofilm formation in Escherichia coli K-12. *Research in Microbiology*, 155(7):514–521, 2004.

[7] Elena Biselli, Severin Josef Schink, and Ulrich Gerland. Slower growth of ¡i¿Escherichia coli¡/i¿ leads to longer survival in carbon starvation due to a decrease in the maintenance rate. *Molecular Systems Biology*, 16(6):e9478, jun 2020.

[8] Regoes Roland R., Wiuff Camilla, Zappala Renata M., Garner Kim N., Baquero Fernando, and Levin Bruce R. Pharmacodynamic Functions: a Multiparameter Approach to the Design of Antibiotic Treatment Regimens. *Antimicrobial Agents and Chemotherapy*, 48(10):3670–3676, oct 2004.

[9] Bauvois Cédric, Ibuka Akiko Shimizu, Celso Almeida, Alba Jimena, Ishii Yoshikazu, Frère Jean-Marie, and Galleni Moreno. Kinetic Properties of Four Plasmid-Mediated AmpC β-Lactamases. *Antimicrobial Agents and Chemotherapy*, 49(10):4240–4246, oct 2005.

[10] M Galleni and J M Frère. A survey of the kinetic parameters of class C β-lactamases. Penicillins. *Biochemical Journal*, 255(1):119–122, oct 1988.

[11] Joseph P Hou and John W Poole. Kinetics and Mechanism of Degradation of Ampicillin in Solution. *Journal of Pharmaceutical Sciences*, 58(4):447–454, apr 1969.

[12] R. Milo, R. Phillips, and N. Orme. *Cell Biology by the Numbers.* Garland Science, Taylor & Francis Group, 2016.

[13] Mary Ann Moran, Brandon Satinsky, Scott M Gifford, Haiwei Luo, Adam Rivers, Leong-Keat Chan, Jun Meng, Bryndan P Durham, Chen Shen, Vanessa A Varaljay, Christa B Smith, Patricia L Yager, and Brian M Hopkinson. Sizing up metatranscriptomics. *The ISME Journal*, 7(2):237–243, 2013.

[14] Fraenkel D G. and Levisohn S R. Glucose and Gluconate Metabolism in an Escherichia coli Mutant Lacking Phosphoglucose Isomerase. *Journal of Bacteriology*, 93(5):1571–1578, may 1967.

[15] Sławomir Wąsik, Michał Arabski, Zuzanna Drulis-Kawa, and Jerzy Gubernator. Laser interferometry analysis of ciprofloxacin and ampicillin diffusion from liposomal solutions to water phase. *European Biophysics Journal*, 42(7):549–558, 2013.

[16] R Höber. *Physical Chemistry of Cells and Tissues.* Churchill, 1947.

[17] Garima Kapoor, Saurabh Saigal, and Ashok Elongavan. Action and resistance mechanisms of antibiotics: A guide for clinicians. *Journal of Anaesthesiology Clinical Pharmacology*, 33(3), 2017.

[18] Reshma P Shetty, Drew Endy, and Thomas F Knight. Engineering BioBrick vectors from BioBrick parts. *Journal of Biological Engineering*, 2(1):5, apr 2008.

[19] Gerard D Wright and Paul R Thompson. Aminoglycoside phosphotransferases: proteins, structure, and mechanism. *FBL*, 4(4):9–21, 1999.

[20] Estrella Rojo-Molinero, María D. Macià, and Antonio Oliver. Social behavior of antibiotic resistant mutants within pseudomonas aeruginosa biofilm communities. *Frontiers in Microbiology*, 10:570, 2019.

[21] Gjonbalaj Mergim, Keith James W., Do Mytrang H., Hohl Tobias M., Pamer Eric G., and Becattini Simone. Antibiotic Degradation by Commensal Microbes Shields Pathogens. *Infection and Immunity*, 88(4):10.1128/iai.00012–20, mar 2020.

[22] G P Bongaerts and G M Kaptijn. Aminoglycoside phosphotransferase-II-mediated amikacin resistance in Escherichia coli. *Antimicrobial Agents and Chemotherapy*, 20(3):344–350, sep 1981.

[23] Giampaolo Pitruzzello, Stephen Thorpe, Steven Johnson, Adrian Evans, Hermes Gadêlha, and Thomas F Krauss. Multiparameter antibiotic resistance detection based on hydrodynamic trapping of individual e. coli. *Lab on a Chip*, 19(8):1417–1426, 2019.
